# Oxidative Stress and Necrotizing Enterocolitis in Preterm Newborns: The Role of GSTM1 and GSTT1 Null Genotypes

**DOI:** 10.3390/biom16060900

**Published:** 2026-06-18

**Authors:** Alexandre Alberto Barros Duarte, Danielle Lopes Teixeira Ferdinando, Vânia Belintani Piatto, Heloísa Cristina Caldas

**Affiliations:** 1 Departamento de Pediatria e Cirurgia Pediátrica, Faculdade de Medicina de São José do Rio Preto (FAMERP), Hospital da Criança e Maternidade (FUNFARME), Avenida Brigadeiro Faria Lima, 5416, São José do Rio Preto, São Paulo CEP-15090-000, Brazil; dralexandre.duarte@gmail.com (A.A.B.D.); danielle.ciperiopreto@gmail.com (D.L.T.F.); 2 Departamento de Anatomia, Faculdade de Medicina de São José do Rio Preto (FAMERP), Avenida Brigadeiro Faria Lima, 5416, São José of Rio Preto, São Paulo CEP-15090-000, Brazil; 3 Departamento de Biologia Molecular, Faculdade de Medicina de São José do Rio Preto (FAMERP), Hospital da Criança e Maternidade (FUNFARME), Avenida Brigadeiro Faria Lima, 5416, São José do Rio Preto, São Paulo CEP-15090-000, Brazil; heloisa@famerp.br

**Keywords:** glutathione transferase, necrotizing enterocolitis, oxidative stress, preterm infants

## Abstract

Necrotizing enterocolitis (NEC) is a multifactorial disease associated with prematurity, intestinal hypoperfusion, dysbiosis, and oxidative stress. Interindividual variability in disease occurrence suggests a role for genetic susceptibility. Null genotypes of the GSTM1 and GSTT1 genes result in absent glutathione S-transferase activity and may impair antioxidant defenses. This study investigated whether GSTM1 and GSTT1 null genotypes are associated with NEC development and severity in preterm newborns. This single-center case–control pilot study included 100 preterm newborns (50 NEC and 50 controls). Genotyping was performed by multiplex polymerase chain reaction. Baseline characteristics were comparable between groups (*p* > 0.05). Stages II-A and II-B accounted for 82% of NEC cases. A significant inverse correlation was observed between gestational age and postnatal age at NEC diagnosis (r = −0.5994; *p* < 0.0001). The GSTM1-null genotype was more frequent in the NEC group (60% vs. 36%) and was associated with increased disease risk in both unadjusted (OR = 2.667; 95%CI: 1.188–5.986; *p* = 0.027) and adjusted analyses (aOR = 3.09; 95%CI: 1.29–7.40; *p* = 0.011). No significant associations were observed for GSTT1, combined genotypes, or disease severity. These findings provide preliminary evidence of an association between the GSTM1-null genotype and NEC susceptibility. Given the exploratory pilot design, these results should be considered hypothesis-generating and require confirmation in larger prospective studies.

## 1. Introduction

Necrotizing enterocolitis (NEC) remains a major challenge in neonatal care, particularly among preterm infants, in whom the disease is associated with substantial morbidity and mortality [1]. Although its clinical relevance is well established, the mechanisms underlying its onset and progression are not completely understood, reflecting a complex relationship between developmental, environmental, and biological factors [2].

Current evidence suggests that intestinal immaturity alone is insufficient to explain the occurrence of NEC [3]. Instead, disease development appears to depend on the interaction between an underdeveloped intestinal barrier, an exaggerated inflammatory response, and external triggers such as enteral feeding and microbial colonization [4,5].

Within this context, oxidative stress has gained increasing attention as a contributing mechanism [6]. The imbalance between the production of reactive oxygen species (ROS) and the limited antioxidant capacity of preterm infants may enhance tissue injury and amplify inflammatory pathways involved in NEC [7,8,9,10,11].

The efficiency of antioxidant defense systems varies among individuals and may be partially determined by genetic factors [12]. Glutathione S-transferases (GSTs) play a critical role in cellular detoxification processes, particularly in neutralizing ROS [13].

Among these enzymes, GSTM1 and GSTT1 are of particular interest due to common gene deletions that result in complete loss of enzymatic activity [14,15,16]. These null genotypes have been associated with increased vulnerability to oxidative stress in several clinical conditions, including neonatal disorders [14,15,16].

In preterm newborns, whose antioxidant defense systems are intrinsically immature, the absence of GST enzymatic activity may further impair the neutralization of reactive oxygen species, contributing to intestinal barrier dysfunction, epithelial injury, and amplification of inflammatory pathways associated with NEC [14,15,16].

In addition, the impact of GSTM1 and GSTT1 null genotypes may be modulated by environmental and clinical factors frequently associated with NEC, such as extreme prematurity, enteral feeding exposure, microbial dysbiosis, hypoxia, and systemic inflammation. The interaction between genetic susceptibility and these external triggers may therefore increase vulnerability to oxidative intestinal injury and contribute to NEC development and progression through gene–environment interactions [13,14,15,16].

Despite the biological plausibility linking oxidative stress and NEC, the contribution of GSTM1 and GSTT1 polymorphisms to the disease has not been adequately explored. Understanding whether these genetic variants influence susceptibility to NEC may provide new insights into its pathophysiology and help identify at-risk populations.

Therefore, this study aimed to evaluate the association between GSTM1 and GSTT1 gene polymorphisms and the occurrence of necrotizing enterocolitis in newborns. In addition, the association between these genotypes, individually and in combination, and disease severity was evaluated.

## 2. Materials and Methods

### 2.1. Study Design

This single-center, case–control pilot study was conducted to investigate the association between GSTM1 and GSTT1 gene polymorphisms and necrotizing enterocolitis (NEC) in 100 newborns with and without the disease, admitted to the Neonatal Unit of the institution between October and December 2025. Participants were selected from a total of 250 neonatal admissions during the study period, based on predefined inclusion and exclusion criteria. Controls consisted of preterm newborns admitted to the neonatal unit during the same study period who did not develop NEC. Eligible controls were selected consecutively according to the predefined inclusion and exclusion criteria of the study. The control group was drawn from the same classification of prematurity represented in the NEC group.

### 2.2. Clinical and Anthropometric Assessment

The modified Bell staging criteria [17,18] were used for the diagnosis of NEC and for inclusion of newborns in the case group.

Newborns from both groups were excluded if they presented congenital malformations involving the cardiac, gastrointestinal, renal, or respiratory systems; laboratory-confirmed congenital infections; sepsis and/or meningitis diagnosed before NEC onset; or if their mothers had a history of STORCH/HIV infections (syphilis, toxoplasmosis, rubella, cytomegalovirus, herpes simplex virus, or human immunodeficiency virus), substance abuse during pregnancy, or use of opioids or respiratory depressant drugs in the peripartum period. Sepsis occurring after the diagnosis of NEC was considered a complication that may arise during the clinical course of disease and, therefore, was not regarded as an exclusion criterion. Newborns whose parents or legal guardians did not provide informed consent were also excluded.

Newborns were classified according to gestational age as follows: late preterm (36 to 37 weeks and 6 days), moderate preterm (31 to 35 weeks and 6 days), and extremely preterm (22 to 30 weeks and 6 days) [19,20]. According to birth weight, newborns were categorized as appropriate birth weight (≥2500 g), low birth weight (1500 to <2500 g), very low birth weight (1000 to <1500 g), and extremely low birth weight (<1000 g) [21].

Additional demographic data included type of delivery (vaginal or cesarean), type of birth (singleton or multiple), and Apgar scores at the 1st and 5th minutes. All participants were from the same geographic region (State of São Paulo, Brazil), in an attempt to reduce population heterogeneity.

### 2.3. Molecular Analysis

Genomic DNA was extracted from peripheral venous blood leukocytes using the Illustra Blood GenomicPrep Mini Spin Kit™ (GE Healthcare, Little, Chalfont, UK), according to the manufacturer’s instructions. DNA samples were initially stored at 4 °C for up to 24 h and subsequently preserved at −20 °C until analysis.

The presence or absence of GSTM1 and GSTT1 gene deletions was determined by multiplex polymerase chain reaction (multiplex PCR), using previously described primers [22].

The GSTM1 positive genotype was identified by the presence of a 273-base pair (bp) fragment, while the GSTT1 positive genotype was identified by a 459 bp fragment. The absence of these amplification products indicated null genotypes for GSTM1 and GSTT1.

The GSTM4 gene (163 bp) was used as an internal control to validate the multiplex PCR reaction, ensuring the integrity of DNA amplification and minimizing the risk of false-negative results.

Accordingly, participants were classified as having either positive or null genotypes for each gene. The multiplex PCR methodology allowed the identification of homozygous deletion (null/null) genotypes for GSTM1 and GSTT1. In the absence of the corresponding amplification product, individuals were classified as carrying the null genotype, whereas the presence of an amplification product indicated a non-null genotype. However, this method does not distinguish homozygous non-deleted individuals from heterozygous carriers and therefore permits classification only as null or non-null genotypes. To assess genotyping reliability, 10% of the samples were randomly selected for repeat analysis. Repeated genotyping demonstrated 100% concordance with the original results, and no genotyping failures were observed.

All molecular analyses were performed blinded to clinical data.

#### 2.3.1. Multiplex PCR Conditions

Multiplex PCR reactions were performed in a final volume of 25 μL containing 200 ng of genomic DNA, 10 pmol of each primer, and OneTaq™ Hot Start Quick-Load™ 2X Master Mix (New England Biolabs, Ipswich, MA, USA), following the manufacturer’s instructions. PCR amplification included an initial denaturation at 94 °C for 3 min, followed by 35 cycles of denaturation at 94 °C for 1 min, annealing at 60 °C for 1 min, and extension at 72 °C for 2 min, with a final extension at 72 °C for 10 min.

Amplified products were separated by electrophoresis on 2.2% agarose gels using the FlashGel™ DNA System (Lonza, Rockland, ME, USA) and visualized using the FlashGel™ Camera (Lonza, Rockland, ME, USA).

#### 2.3.2. Combined Genotype Analysis

Combined genotypes were categorized into four groups: GSTM1-null/GSTT1-null, GSTM1-null/GSTT1-positive, GSTM1-positive/GSTT1-positive, and GSTM1-positive/GSTT1-null. These combinations were analyzed to explore potential gene-gene interactions and their association with susceptibility to NEC and disease severity.

### 2.4. Ethical Considerations

This study was conducted in accordance with the Declaration of Helsinki and was approved by the Ethics Committee of the Medical School of São José do Rio Preto, SP, Brazil (Protocol No. 6.951.562, July 2024). Written informed consent was obtained from the parents or legal guardians of all participants prior to enrollment.

### 2.5. Statistical Analysis

Statistical analyses were initially performed using descriptive methods to assess data distribution. The normality of continuous variables was evaluated using the Kolmogorov–Smirnov test.

Categorical variables are presented as absolute and relative frequencies (%), while continuous variables are expressed as mean ± standard deviation (SD) for normally distributed data or as median (min–max) for non-normally distributed data.

Comparisons between independent groups were performed using the unpaired Student’s *t*-test for normally distributed variables and the Mann–Whitney U test for non-normally distributed variables.

Categorical variables were compared using the chi-square (χ^2^) test or Fisher’s exact test, as appropriate. For contingency tables larger than 2 × 2 with low expected frequencies, the Fisher–Freeman–Halton extension of Fisher’s exact test was applied.

Pearson’s correlation coefficient was used to assess the relationship between gestational age and postnatal age at NEC diagnosis.

Odds ratios (OR) with 95% confidence intervals (95% CI) were calculated to estimate associations between variables. In addition, a multivariable logistic regression analysis was performed to evaluate the independent association between the GSTM1 genotype and NEC after adjustment for GSTT1 genotype, gestational age, birth weight, and sex. Adjusted odds ratios (aORs) and their respective 95% confidence intervals were calculated.

A two-tailed *p*-value < 0.05 was considered statistically significant.

Univariable analyses were performed using GraphPad InStat software, version 3.00 (GraphPad Software Inc., San Diego, CA, USA). Multivariable logistic regression analyses were performed using IBM SPSS^®^ Statistics for Windows software, version 19 (IBM Corp., Armonk, NY, USA).

No adjustment for multiple comparisons was performed due to the exploratory nature of the study; therefore, the results should be interpreted with caution.

## 3. Results

### 3.1. Clinical and Demographic Characteristics of the Study Population

A total of 100 newborns were enrolled, including 50 with NEC and 50 controls.

Baseline characteristics of both groups are shown in Table 1. No statistically significant differences were observed between the case and control groups in terms of gestational age, birth weight, Apgar scores, sex, or frequency of extremely low birth weight (all *p* > 0.05), indicating that the groups were comparable.

In the NEC group, 17 (34%) required surgical management. Among these, more severe disease stages were more frequent, particularly the Bell stages II-B and III-B, observed in 6 (35%) and 8 (47%) patients, respectively. Conservative treatment was sufficient in 66% of cases, and overall survival was 86%.

A significant inverse correlation was identified between gestational age at birth and postnatal age at the time of NEC diagnosis (r = −0.5994; *p* < 0.0001).

### 3.2. Result of Molecular Analysis

Genotyping was performed using multiplex PCR, with successful amplification confirmed by agarose gel electrophoresis (Supplementary Figure S1).

Genotype distributions are presented in Table 2. The GSTM1-null genotype was significantly more frequent in the case group (60% vs. 36%) and was associated with an increased risk of NEC (OR = 2.667; 95% CI: 1.188–5.986; *p* = 0.027). In contrast, no significant association was observed for GSTT1 (*p* > 0.05). Combined genotype analysis did not demonstrate a significant association with NEC (all *p* > 0.05), although the relatively small sample size may have limited the statistical power to detect modest effects. Additionally, neither GSTM1 nor GSTT1 genotypes, individually or in combination, were significantly associated with severe NEC (stage III-B) among cases (all *p* > 0.05). Given the limited number of severe cases, these findings should be interpreted cautiously, as the analyses may have been underpowered to detect modest associations.

To further evaluate whether the association between GSTM1 and NEC was independent of potential confounding factors, a multivariable logistic regression analysis was performed, including GSTM1 genotype, GSTT1 genotype, gestational age, birth weight, and sex. The GSTM1-null genotype remained independently associated with NEC (aOR = 3.09, 95% CI: 1.29–7.40, *p* = 0.011). No significant independent associations were observed for GSTT1 genotype (*p* = 0.803), gestational age (*p* = 0.449), birth weight (*p* = 0.064), or sex (*p* = 0.719).

## 4. Discussion

Beyond established clinical risk factors, the heterogeneous presentation of necrotizing enterocolitis suggests that additional biological mechanisms may contribute to disease susceptibility [23,24]. In this context, interindividual variability in host responses has emerged as a relevant area of investigation, particularly regarding pathways involved in inflammation and oxidative stress [25,26,27]. Increasing attention has therefore been directed toward host-related factors, including genetic variability, as potential modulators of individual susceptibility to NEC [28,29].

In the present pilot study, the similarity between the Case and Control groups across key neonatal variables supports the comparability of the study groups. However, because several established clinical risk factors for NEC, including feeding-related and treatment-related variables, were not systematically collected, residual confounding cannot be completely excluded. This observation is consistent with previous reports indicating that prematurity and low birth weight, although central to NEC risk, are insufficient to explain its heterogeneous clinical presentation [4,5,23,24].

Furthermore, the inverse correlation observed between gestational age and postnatal age at NEC diagnosis is consistent with previous clinical observations indicating that more premature infants tend to develop NEC at a later postnatal age [4,5,23,24]. This finding should be interpreted primarily as a descriptive clinical observation and does not provide direct mechanistic insight into NEC pathogenesis.

The main finding of this study was the observed association between the GSTM1-null genotype and an increased likelihood of NEC. From a mechanistic perspective, the absence of GSTM1 enzymatic activity may impair the detoxification of reactive oxygen species, thereby amplifying oxidative injury and inflammatory signaling within the immature intestine [30]. This interpretation aligns with current models of NEC pathophysiology, in which oxidative stress plays a central role in epithelial injury and disease progression [4,7,10,11,31,32].

Preterm infants are characterized by immature antioxidant defense systems, rendering them particularly vulnerable to oxidative imbalance [6,7,8,9,10,11]. The accumulation of reactive oxygen species may exacerbate intestinal inflammation through pathways such as Toll-like receptor 4 (TLR4), which has been implicated in NEC development [15,33,34,35,36,37]. Within this framework, GSTM1 deletion may represent an additional layer of susceptibility, potentially lowering the threshold for intestinal injury in response to environmental triggers [8,9,25,26,27,28,29,30,31,38]. Importantly, the association between the GSTM1-null genotype and NEC remained significant after adjustment for gestational age, birth weight, sex, and GSTT1 genotype. Although this finding was independent of these measured neonatal characteristics, residual confounding remains a relevant concern because several established clinical risk factors for NEC were not systematically collected. Consequently, the observed association should be interpreted as preliminary and with appropriate caution. Nevertheless, the persistence of the association after multivariable adjustment is consistent with the hypothesis that GSTM1-related antioxidant defense mechanisms may contribute to individual susceptibility to NEC.

In contrast, no association was observed for the GSTT1-null genotype, suggesting that the contribution of glutathione S-transferase isoforms to oxidative defense may be functionally distinct. Similarly, combined genotype analysis did not yield statistically significant results, although the distribution pattern—characterized by a greater frequency of GSTM1-null combinations in cases—remains biologically consistent with impaired antioxidant capacity [39,40,41,42]. The lack of significant associations for GSTT1 and combined genotypes should be interpreted cautiously, as these analyses were based on relatively small subgroups and may have been underpowered to detect modest effects. Consequently, the possibility of clinically relevant associations cannot be excluded.

Differences between GSTM1 and GSTT1 findings may also reflect functional distinctions among glutathione S-transferase isoforms, which exhibit different substrate specificities and may contribute unequally to antioxidant defense pathways. In addition, population-specific genetic backgrounds, environmental exposures, and gene–environment interactions may influence the magnitude and direction of observed associations [39,40,41,42]. Therefore, the absence of significant associations for GSTT1 and combined genotypes in the present study should not necessarily be interpreted as evidence that these variants are biologically irrelevant, but rather as findings that require confirmation in larger and more diverse populations.

No significant associations were identified between GST genotypes and disease severity, as defined by Bell stage III-B NEC. This finding should also be interpreted cautiously because the number of severe cases was limited. Although this may reflect insufficient statistical power, it may also indicate that genetic susceptibility and disease progression are influenced by partially distinct mechanisms. Indeed, NEC severity is likely modulated by additional factors, including inflammatory cascades, microbiota composition, and clinical management variables [2,4,5].

Notably, no previous studies have directly evaluated the role of GSTM1 and GSTT1 polymorphisms in NEC, underscoring the novelty of the present findings. However, indirect evidence from other neonatal conditions associated with oxidative stress—such as retinopathy of prematurity, intraventricular hemorrhage, periventricular leukomalacia, and bronchopulmonary dysplasia—supports an association between GSTM1-null genotypes and increased disease risk or severity [8,9,14,16,25,26,27,28,29,30].

An important limitation of this study is that several established clinical risk factors for NEC, including feeding-related practices, medication exposures, cardiorespiratory support requirements, and other neonatal clinical variables, were not systematically collected. Therefore, their potential confounding or modifying effects on the observed associations between GST genotypes and NEC could not be evaluated.

Several additional limitations should also be acknowledged, including the relatively small sample size, the evaluation of only two genes within the antioxidant system, and the use of multiplex PCR for genotyping. While this method reliably identifies deletion (null) and non-deletion genotypes, it does not distinguish heterozygous from homozygous non-deleted carriers. Furthermore, despite multivariable adjustment for gestational age, birth weight, sex, and GSTT1 genotype, the exploratory nature of this pilot study and the absence of adjustment for multiple comparisons warrant cautious interpretation of the results. Nevertheless, the findings suggest that genetic variants related to the antioxidant response may influence susceptibility to NEC.

Thus, further studies with larger sample sizes and integrative approaches—including genomic, functional, and environmental analyses—are needed to confirm these findings and to better elucidate the underlying mechanisms. The future integration of such biomarkers into clinical practice may contribute to risk stratification strategies and support the advancement of precision medicine in neonatal clinical and surgical care.

## 5. Conclusions

The GSTM1-null genotype was associated with an increased risk of necrotizing enterocolitis in preterm newborns. In contrast, the GSTT1-null genotype and combined genotypes were not significantly associated with disease occurrence or severity.

Negative findings regarding GSTT1, combined genotypes, and disease severity should be interpreted cautiously, as the study may have been underpowered to detect modest associations.

These findings provide preliminary and exploratory evidence of an association between the GSTM1-null genotype and susceptibility to necrotizing enterocolitis. Although this association remained significant after multivariable adjustment, including GSTT1 genotype, gestational age, birth weight, and sex, the study design is pilot in nature, with a limited sample size and no adjustment for multiple comparisons. Accordingly, the results should be interpreted cautiously and considered hypothesis-generating, warranting confirmation in larger, adequately powered prospective studies.

## Figures and Tables

**Table 1:** Baseline characteristics of the study population.

Variable	Case (*n* = 50)	Control (*n* = 50)	*p*-Value
Gestational age, weeks median (min–max)	29 (24–37)	29 (23–36)	0.353 *
Birth weight, g median (min–max)	1267.5 (600–3700)	1050 (500–3725)	0.095 *
Apgar score (1 min) median (min–max)	7 (2–9)	6 (4–9)	0.176 *
Apgar score (5 min) median (min–max)	9 (7–10)	9 (7–10)	0.059 *
Male sex, *n* (%)	27 (54)	28 (56)	1.000 ^†^
Extremely low birth weight (<1000 g), *n* (%)	15 (30)	20 (40)	0.172 ^†^
Cesarean delivery, *n* (%)	38 (76)	37 (74)	1.000 ^†^

Data are presented as median (min–max) or *n* (%). *n*, number; g, grams. * Mann–Whitney U test. ^†^ Fisher’s exact test.

**Table 2:** Association of GSTM1 and GSTT1 genotypes with necrotizing enterocolitis.

Genotype	Case (*n* = 50)	Control (*n* = 50)	OR (95% CI)	*p*-Value
GSTM1				
Null	30 (60)	18 (36)	2.667 (1.188–5.986)	**0.027**
Positive	20 (40)	32 (64)	Reference	
GSTT1				
Null	10 (20)	9 (18)	1.139 (0.419–3.098)	1.000
Positive	40 (80)	41 (82)	Reference	
Combined genotypes				
GSTM1 [−]/GSTT1 [−]	9 (18)	5 (10)	1.976 (0.612–6.382)	0.388
GSTM1 [−]/GSTT1 [+]	21 (42)	13 (26)	2.061 (0.885–4.801)	0.139
GSTM1 [+]/GSTT1 [+]	19 (38)	28 (56)	Reference	
GSTM1 [+]/GSTT1 [−]	1 (2)	4 (8)	0.235 (0.025–2.179)	0.362

Bold value is statistically significant (*p* < 0.05). OR: odds ratio; CI: confidence interval; *n*: number. [−]: null genotype; [+]: positive genotype.

## Data Availability

The data presented in this study are available on request from the corresponding author due to ethical and privacy considerations.

## Supplementary Materials

The following supporting information can be downloaded at: https://www.mdpi.com/article/10.3390/biom16060900/s1, Figure S1. Representative multiplex PCR analysis of GSTM1 and GSTT1 genotypes resolved on a 2.2% agarose gel. The 163 bp fragment corresponds to the GSTM4 gene and was used as an internal amplification control. The 273 bp and 459 bp fragments indicate the presence of the GSTM1 and GSTT1 genes, respectively. Representative genotypes are shown as follows: GSTM1-positive/GSTT1-positive (lanes 1, 4, 9, and 11); GSTM1-null/GSTT1-positive (lanes 2, 3, and 7); GSTM1-null/GSTT1-null (lanes 5, 6, and 8); and GSTM1-positive/GSTT1-null (lanes 10 and 12). MM: molecular weight marker (FlashGel DNA Marker 50–1500 bp, Lonza™). bp: base pairs.

## Abbreviations

The following abbreviations are used in this manuscript:

aOR	Adjusted Odds Ratio
aORs	Adjusted Odds Ratios
NEC	Necrotizing Enterocolitis
ROS	Reactive Oxygen Species
GSTs	Glutathione S-transferases
GSTM1	Glutathione S-transferase Mu 1
GSTM4	Glutathione S-transferase Mu 4
GSTT1	Glutathione S-transferase Theta 1
OR	Odds Ratio
CI	Confidence Interval
PCR	Polymerase Chain Reaction
DNA	Deoxyribonucleic acid
