# Supplementary material for: Oxidative Stress and Necrotizing Enterocolitis in Preterm Newborns: The Role of GSTM1 and GSTT1 Null Genotypes
Source: Biomolecules. 2026 Jun 18;16(6):900. doi: 10.3390/biom16060900 (PMC13297310; doi:10.3390/biom16060900)
Supplement: Supplementary file 1 [file biomolecules-16-00900-s001.zip › biomolecules-4362387-supplementary/biomolecules-4362387-supplementary.pdf]

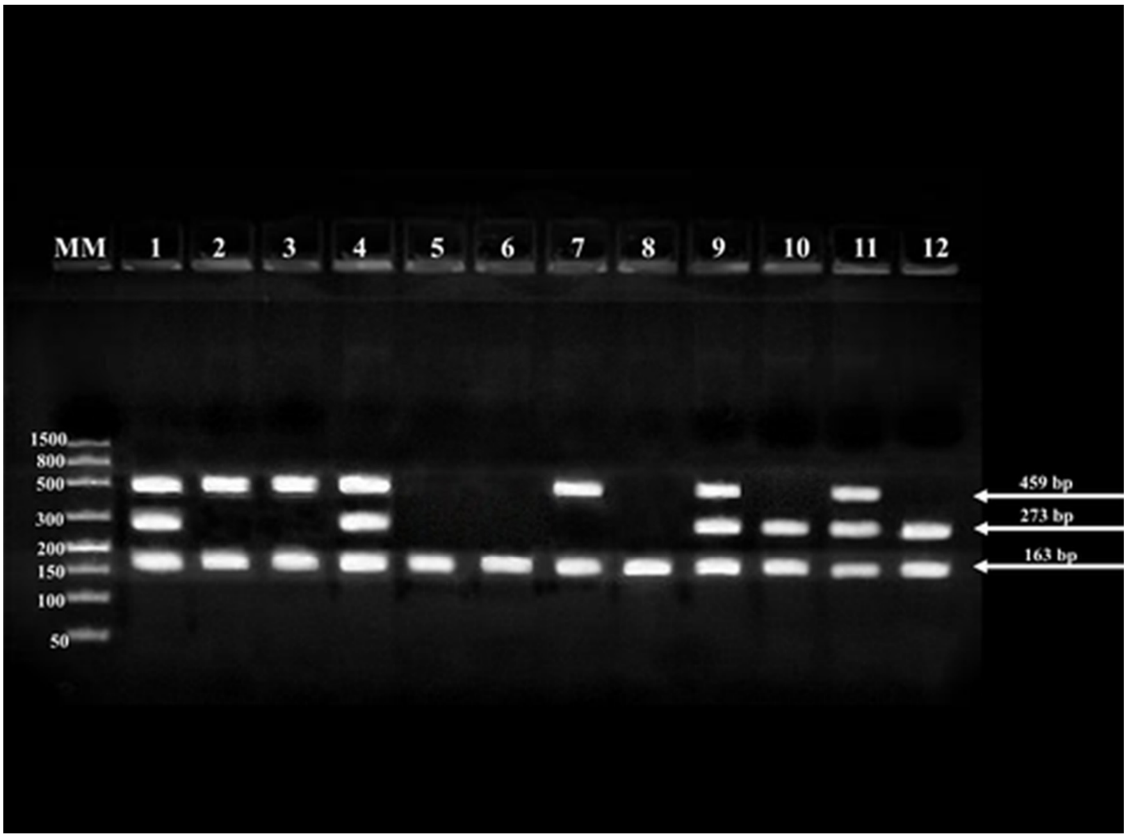


**Figure S1.** Representative multiplex PCR analysis of GSTM1 and GSTT1 genotypes resolved on a 2.2% agarose gel. The 163 bp fragment corresponds to the GSTM4 gene and was used as an internal amplification control. The 273 bp and 459 bp fragments indicate the presence of the GSTM1 and GSTT1 genes, respectively. Representative genotypes are shown as follows: GSTM1-positive/GSTT1-positive (lanes 1, 4, 9, and 11); GSTM1-null/GSTT1-positive (lanes 2, 3, and 7); GSTM1-null/GSTT1-null (lanes 5, 6, and 8); and GSTM1-positive/GSTT1-null (lanes 10 and 12). MM: molecular weight marker (FlashGel DNA Marker 50–1500 bp, Lonza™). bp: base pairs.
